# Health professionals: leaving their countries to migrate to the United States and Canada

**DOI:** 10.1186/1472-6963-14-S2-P71

**Published:** 2014-07-07

**Authors:** Christine Mahoney

**Affiliations:** 1 Management Dept, College of Business, Minnesota State University, Mankato, MN 56001, USA

## Background

The United States and Canada are experiencing an increased demand for licensed health professionals; currently both have shortages and maldistribution of licensed health professionals. The factor having the greatest impact in creating the shortage is the increased demand for services due to an aging population. The demand for technological advancements and too few licensed health professionals being trained also contribute to the increased demand. The health professional shortage is forecast to last at least 15 years. Neither country’s educational pipeline currently projects enough new graduates to meet the demand. Simultaneously, a majority of countries around the world also are experiencing a shortage and can ill afford to lose any of their licensed health professionals. However, health professionals from other countries continue to be relied on to meet part of the demand.

## Materials and methods

As part of a larger European Union based project, an online survey collected information from over 500 licensed health professionals who have migrated to either the United States or Canada. Links to the survey were placed on web sites of health professional organizations. The survey was open to respondents for sixty days.

## Results

Survey results reveal that the reason health professionals come to the United States and Canada are as follows: better working conditions (US 88%, CA 78%), higher earnings (35%, 42%),better employment benefits (70%, 30%), job growth opportunities (60%, 51%), training and educational advancement (74%, 60%), resources and advanced technologies available to complete job tasks (46%, 34%), improved lifestyle conditions (86%, 100%), political stability (46%, 52%), friends or family live there (66%, 38%). Data was also collected on reasons for leaving home country.

## Conclusions

Numerous issues should be addressed that create difficulties for immigrant health professionals. These include, but are not limited to, getting licenses approved, partnering with foreign universities to train health professionals, and providing educational grants for immigrants at universities (in US or Canada). Additional strategies that must be employed are increasing the number of health professionals trained, effectively using advanced technologies such as telemedicine, and task shifting among health professionals. While doing so, both countries must make every effort to adhere to the World Health Organization’s Code of Practice on the International Recruitment of Health Personnel.

